# Editorial: Preclinical animal models and measures of pain: improving predictive validity for analgesic drug development - volume II

**DOI:** 10.3389/fpain.2024.1523938

**Published:** 2025-01-07

**Authors:** Anke Tappe-Theodor, Thomas J. Martin, S. Stevens Negus

**Affiliations:** ^1^ Institute of Pharmacology, University of Heidelberg, Heidelberg, Germany; ^2^ Pain Mechanisms Laboratory, Department of Anesthesiology, Wake Forest University School of Medicine, Winston-Salem, NC, United States; ^3^ Department of Pharmacology and Toxicology, Virginia Commonwealth University, Richmond, VA, United States

**Keywords:** animal models, behavioral tests, predictive validity, analgesic drugs, pain measurement

**Editorial on the Research Topic**
Preclinical animal models and measures of pain: improving predictive validity for analgesic drug development - volume II

Chronic pain affects millions worldwide, thereby severely impacting the quality of life, and effective pain management still remains one of the most significant challenges in modern medicine. Despite the urgent need for better treatments, the development of new analgesic drugs has progressed slowly, with many promising candidates failing in clinical trials (1–4). A major reason for this high attrition rate is the disconnect between preclinical models and their predictive power in clinical settings (5, 6). Nonetheless, preclinical animal models are crucial in the drug development process, offering mechanistic insights and serving as an important platform for efficacy and safety testing of new treatments. However, their limitations in accurately mimicking human pain experiences have led to a critical bottleneck in the identification of effective analgesics.

Rodent models have been used to study pain pathways and evaluate potential treatments, with nociceptive tests such as the hot-plate and tail-flick assays widely employed to assess acute pain responses. However, these reflex-based models do not adequately capture the complexity of either acute or chronic pain, which often involves emotional, cognitive, and sensory components (7, 8). Chronic pain conditions like fibromyalgia or neuropathic pain are characterized by persistent pain along with emotional disturbances and cognitive impairments (9), aspects often overlooked in preclinical research. This disconnect has led to a recognition that more complex models, which account for the multidimensional nature of pain, are necessary.

Accordingly, recent advances in pain research focused on more sophisticated methods and models integrating both sensory and affective dimensions of pain to address the negative impact of pain on function and quality of life, and enhance the predictive validity of animal studies for human clinical outcomes (10–12).

A key aspect of advancing preclinical pain models involves refining behavioral endpoints that more closely mirror the affective and motivational components of human pain (13–16). Chronic pain in humans often leads to symptoms such as reduced physical activity, social withdrawal, and depressive-like behavior—features now being incorporated into animal models (17). Recognizing the limitations of traditional nociceptive measures, researchers have begun to explore novel behavioral assays that better represent pain-related disability and the broader impact of chronic pain on behavior. Such refinements are critical for improving the assessment of drug efficacy, particularly when considering treatments aimed at addressing not just the sensory, but also the emotional and cognitive consequences of pain.

In this second special issue, we present four studies that explore different preclinical models and methodologies aimed at improving our understanding of pain and enhancing the predictive validity of animal models for analgesic drug development. These studies cover a range of innovative approaches, from exploring the efficacy of combined therapies for fibromyalgia, to evaluating novel behavioral assays for pain-related depression, to investigating how vascular changes in the spinal cord affect drug delivery. Each study provides valuable insights into the complex dynamics of pain and offers potential pathways for improving the translational success of new treatments.

The study by Argenbright et al. investigates the efficacy of pregabalin and hyperbaric oxygen therapy (HBO2) in a rat model of fibromyalgia (FM). FM is a chronic, widespread pain disorder, characterized by both sensory abnormalities and a variety of cognitive and affective comorbidities, including anxiety and depression (18). Preclinical models of FM are vital for understanding these complex interactions, as well as for testing the effectiveness of potential therapies. In this study, the authors used an acidic saline injection model, which mimics the hyperalgesia commonly seen in FM patients, and assessed both sensory (mechanical pain thresholds) and affective components (anxiety-like behaviors). Interestingly, while pregabalin, a known treatment for FM, reduced avoidance behaviors, neither it nor HBO2 significantly alleviated mechanical hyperalgesia or anxiety in this model. These findings highlight the challenges of modeling the affective dimensions of FM and underscore the need for further refinements in preclinical FM models to capture the full spectrum of symptoms experienced by patients.

Building on the theme of novel behavioral endpoints, Santos et al. explore the use of climbing behavior in mice as a tool for assessing pain-related behavioral depression. Traditional models often focus on reflexive responses to painful stimuli, but these fail to account for the motivational deficits associated with chronic pain (19). Santos et al. introduced a climbing assay as a way to measure motivated behavior in mice, hypothesizing that pain would lead to reduced climbing, which could then be reversed by effective analgesic treatments. Their results demonstrated that climbing behavior was depressed by intraperitoneal injections of lactic acid, a well-established model for inducing acute pain in rodents. Ketoprofen, a non-steroidal anti-inflammatory drug (NSAID), effectively reversed this depression, while opioid treatments like fentanyl disrupted climbing behavior in the absence of pain. This provides a critical tool for differentiating between analgesic and sedative effects, which is essential for developing better pain treatments.

Another critical aspect of pain research is understanding how systemic factors, such as vascular health, impact the efficacy of pain treatments. Lobo et al. address this issue in their study by investigating how spinal cord vascular degeneration impairs the penetration and efficacy of duloxetine, a serotonin-norepinephrine reuptake inhibitor commonly used to treat chronic pain conditions like neuropathy and fibromyalgia (20). Using a rodent model of spinal cord vascular degeneration, they found that reduced blood flow in the spinal cord was associated with lower concentrations of duloxetine in the tissue, leading to diminished analgesic effects. This study highlights the importance of considering vascular health when evaluating drug efficacy, particularly for conditions where spinal cord pathology may play a role. The findings suggest that enhancing vascular perfusion could be a potential strategy for improving the delivery and effectiveness of analgesic drugs in patients with spinal cord-related pain disorders.

Finally, the study by Negus et al. explores the role of mu-opioid receptor (MOR) agonist efficacy in determining the balance between analgesic effectiveness and safety. Opioids are among the most potent pain relievers available, but their use is limited by serious side effects, including respiratory depression, motor impairment, and gastrointestinal issues (21). Negus et al. used a novel assay of pain-depressed locomotor behavior in mice to evaluate the analgesic and side-effect profiles of opioids with varying MOR efficacies. Their results showed that intermediate-efficacy MOR agonists, such as buprenorphine, provided effective pain relief with fewer side effects compared to high-efficacy agonists like fentanyl. This suggests that intermediate-efficacy agonists may offer a promising path forward for developing safer opioid therapies, particularly in managing chronic pain conditions. Additionally, the use of pain-depressed behavior as an endpoint highlights the importance of integrating functional measures into preclinical models to assess both the efficacy and safety of analgesic drugs more holistically.

In conclusion, the studies presented in this special issue provide significant advancements in preclinical pain research. By refining animal models and exploring novel behavioral endpoints, these studies contribute to improving the predictive validity of preclinical models for analgesic drug development. The findings underscore the complexity of chronic pain and the need for multidimensional approaches in both modeling pain and assessing potential treatments. As we continue to develop more sophisticated models and methodologies, these advancements will be crucial in guiding the development of more effective and safer treatments for chronic pain patients, bridging the gap between preclinical research and clinical application.
